# Insulin-like growth factor binding protein 2 promotes ovarian cancer cell invasion

**DOI:** 10.1186/1476-4598-4-7

**Published:** 2005-02-02

**Authors:** Eun-Ju Lee, Cristian Mircean, Ilya Shmulevich, Huamin Wang, Jinsong Liu, Antti Niemistö, John J Kavanagh, Je-Ho Lee, Wei Zhang

**Affiliations:** 1 Departments of Pathology, The University of Texas M. D. Anderson Cancer Center, 1515 Holcombe Blvd, Houston, Texas, 77030, USA; 2 Department of Gynecologic Medical Oncology, The University of Texas M. D. Anderson Cancer Center, 1515 Holcombe Blvd, Houston, Texas, 77030, USA; 3 Molecular Therapy Research Center, Samsung Medical Center, Seoul, 135–710, Korea; 4 Institute of Signal Processing, Tampere University of Technology, Tampere, Finland

## Abstract

**Background:**

Insulin-like growth factor binding protein 2 (IGFBP2) is overexpressed in ovarian malignant tissues and in the serum and cystic fluid of ovarian cancer patients, suggesting an important role of IGFBP2 in the biology of ovarian cancer. The purpose of this study was to assess the role of increased IGFBP2 in ovarian cancer cells.

**Results:**

Using western blotting and tissue microarray analyses, we showed that IGFBP2 was frequently overexpressed in ovarian carcinomas compared with normal ovarian tissues. Furthermore, IGFBP2 was significantly overexpressed in invasive serous ovarian carcinomas compared with borderline serous ovarian tumors. To test whether increased IGFBP2 contributes to the highly invasive nature of ovarian cancer cells, we generated IGFBP2-overexpressing cells from an SKOV3 ovarian cancer cell line, which has a very low level of endogenous IGFBP2. A Matrigel invasion assay showed that these IGFBP2-overexpressing cells were more invasive than the control cells. We then designed small interference RNA (siRNA) molecules that attenuated IGFBP2 expression in PA-1 ovarian cancer cells, which have a high level of endogenous IGFBP2. The Matrigel invasion assay showed that the attenuation of IGFBP2 expression indeed decreased the invasiveness of PA-1 cells.

**Conclusions:**

We therefore showed that IGFBP2 enhances the invasion capacity of ovarian cancer cells. Blockage of IGFBP2 may thus constitute a viable strategy for targeted cancer therapy.

## Background

Ovarian cancer is the most lethal gynecological malignancy. Indeed, epithelial ovarian cancer is detected at a late clinical stage in as much as 75% of patients, in whom the overall survival rate is a dismal 14–30% [1]. Hindering the development of effective treatments for the cancer is the fact that the molecular events responsible for the biological behavior of ovarian cancer are poorly understood. Implicated in both ovarian tumorigenesis and physiological follicular proliferation are the insulin-like growth factor I (IGF-I) and IGF-II systems. IGFs are regulated by at least six members of the IGF binding protein (IGFBP) family. High levels of IGFBP2 were detected in the serum or cystic fluid from patients with ovarian cancer compared with those with benign and borderline tumors [2-4]. A recent study further showed that IGFBP2 mRNA was overexpressed to a greater extent in advanced-stage serous ovarian cancer than normal ovarian tissue [5]. In addition, the increase in IGFBP2 expression was found to correlate positively with the levels of the serum tumor marker CA125 [4]. The progression-free interval and overall survival have also proved to be significantly shorter in patients with a high serum level of IGFBP2 at diagnosis than in those with lower levels [6]. Taken together, these data suggest an important role for IGFBP2 in the biology of ovarian cancer.

IGFBP2 is also overexpressed in a wide spectrum of other cancers, including glioma, prostate cancer, synovial sarcoma, neuroblastoma, colon cancer, adrenocortical cancer, lung cancer, Wilms' tumor, and hepatoblastoma [7-17]. The overexpression of IGFBP2 also correlates with the aggressiveness of some tumors, including prostate cancer, hepatoblastoma and glioma [10,17,18], suggesting that IGFBP2 possesses a carcinogenic property. The observation that IGFBP2 has an RGD motif suggests that IGFBP2 modulates the integrin/cytoskeleton system. Indeed, IGFBP2 was recently found to interact with the alpha v beta 3 and alpha 5 beta 1 integrins [19,20]. In addition, IGFBP2 has been found to stimulate the growth of prostate cancer cells, an effect that can be blocked by MAP-kinase and PI3-kinase inhibitors [21]. IGFBP2 was also found to be co-expressed with the vascular endothelial growth factor in pseudopalisading glioma cells surrounding tumor necrosis [22]. Further, IGFBP2 enhances glioma cell invasion by increasing invasion-related genes including MMP2 [23]. These findings collectively suggest that IGFBP2 plays a key role in human cancer development.

In this study, we found that overexpression of IGFBP2 enhanced the invasiveness of ovarian cancer cells. Further, attenuation of IGFBP2 expression by siRNA reduced the invasiveness of ovarian cancer cells.

## Results and Discussion

### IGFBP2 is associated with invasive epithelial ovarian carcinoma

**Figure 1:**
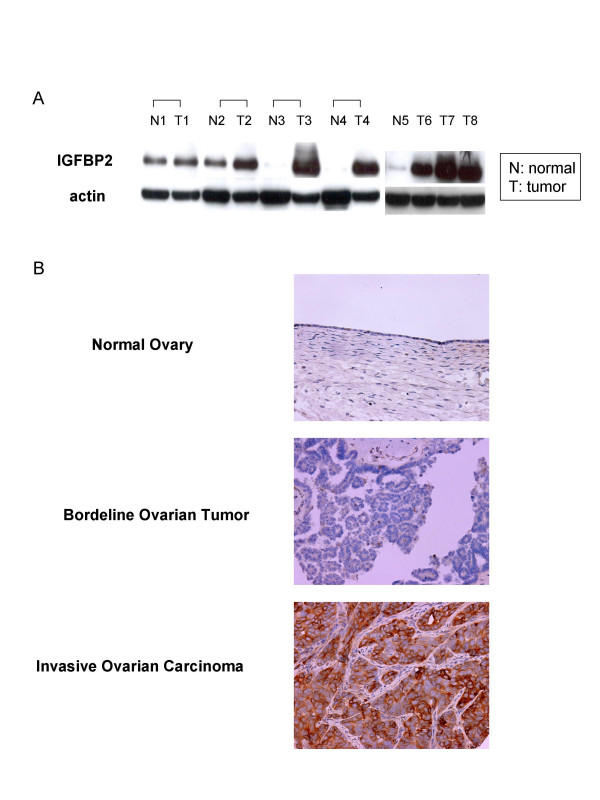
Overexpression of IGFBP2 in ovarian carcinoma. (A) Western blotting analysis in paired normal and cancer tissues (N1 to T4), one normal ovarian (N5) and 3 unpaired ovarian cancer tissues (T6 to T8) showed frequent overexpression of IGFP2 in ovarian cancer tissues compared with normal ovarian tissues. (N: normal ovarian tissue, T: ovarian cancer tissue). (B) Expression of IGFBP2 in normal ovary (100×), borderline ovarian tumor (100×), and invasive ovarian carcinoma (200×) using tissue microarray. IGFBP2 was expressed at greater level in invasive ovarian carcinoma than normal and borderline ovarian tumors.

We first performed western blotting analysis using frozen tissue specimens, which consisted of four paired normal and carcinomatous ovarian tissues, one normal ovarian tissue, and three unpaired ovarian carcinoma tissues. This result showed that IGFBP2 was frequently overexpressed in ovarian cancer tissues compared with normal ovarian tissues (Figure 1A). We then compared the expression of IGFBP2 in normal ovaries, borderline serous ovarian tumors, and invasive serous ovarian carcinomas using a tissue microarray, which showed that IGFBP2 was expressed at significantly different levels between normal tissues and borderline tumors (*p *= 0.03) and between borderline tumors and invasive carcinomas (*p *= 0.03) (Figure 1B, Table 1). Because borderline ovarian tumors have no obvious stromal invasion or infiltrative growth in contrast with invasive ovarian carcinoma [24], this finding suggests that IGFBP2 overexpression could induce invasion nature of ovarian cancer.

**Table 1:** Increasing expression of IGFBP2 with progression of ovarian serous tumors*.

Tissue	IGFBP2 expression (Scores)				*P *value	
		
	3	2	1	0		
Normal Ovaries			◇	◇◇◇◇◇	0.03^#^	
Borderline Ovarian Tumors			◇◇◇◇	◇		
		◇◇	◇◇◇◇◇	◇◇◇◇◇		
Invasive Ovarian Carcinomas	◇◇					0.03^§^
	◇◇◇◇◇					
	◇◇◇◇◇	◇◇◇◇		◇◇◇◇		
	◇◇◇◇◇	◇◇◇◇◇	◇◇◇◇◇	◇◇◇◇◇		

*Each diamond represents one case (Mann-Whitney U test).

^#^*P *value between normal ovaries and borderline ovarian tumors

^§^*P *value between borderline ovarian tumors and invasive ovarian carcinomas

### IGFBP2 enhances the invasiveness of ovarian cancer cells

**Figure 2:**
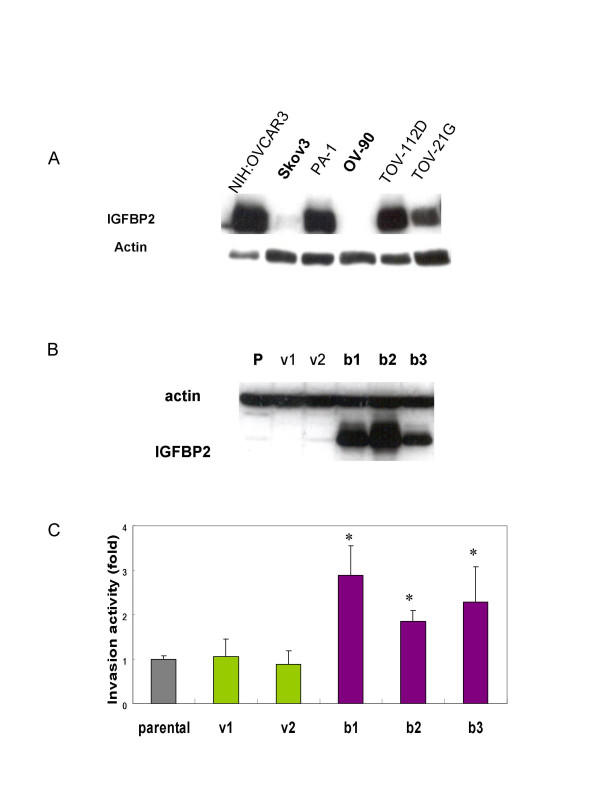
IGFBP2 overexpressing promotes ovarian cancer cell invasion. (A) IGFBP2 expression of six ovarian cancer cell lines. The western blotting analysis shows that the expression level of IGFBP2 is heterogeneous in cell lines. SKOV3 and OV-90 ovarian cancer cell lines have very low endogenous IGFBP2. NIH:OVCAR3, PA-1 and TOV-112D have high levels of IGFBP2 expression whereas TOV-21G has relatively moderate expression of IGFBP2. (B) Two vector transfected clones and three IGFBP2 stable clones with different expression level were obtained. The expression of IGFBP2 was determined by western blotting analysis (p: parental SKOV3 cell line, v: vector transfected cell lines, b: IGFBP2 stable cell lines). (C) The invasion capacity of stable clones showed that IGFBP2 overexpressing cells have invasion potential as 1.84 – 2.89 fold as parental and vector transfected cells. (*p < 0.05)

Thus far, the biological or pathophysiological role of IGFBP2 in ovarian cancer is unknown. To elucidate the role of increased IGFBP2 in ovarian cancer, we therefore generate IGFBP2-overexpressing cells. To obtain suitable cells for this study, we first examined the endogenous expression of IGFBP2 in six ovarian cancer cell lines: NIH:OVCAR3, SKOV3, PA-1, OV-90, TOV-112D, and TOV-21G. IGFBP2 was expressed at different levels in all six cell lines (Figure 2A). Both SKOV3 and OV-90 cells showed very low levels of endogenous IGFBP2; NIH:OVCAR3, PA-1, and TOV-112D cells expressed IGFBP2 at high levels; and TOV-21G cells expressed IGFBP2 at a relatively moderate level. We thus selected SKOV3 cell line and generated two vector transfected clones and three IGFBP2-overexpressing clones by transfection (Figure 2B).

We studied the cellular phenotype of IGFBP2-overexpressing cell lines. Similar to observations in gliomas [23], we did not observe differences in cell proliferation (data not shown). This is contrary to the findings in prostate cancer, adenocortical cancer and neuroblastoma, in which IGFBP2 has been found to stimulate cell proliferation [21,25,26]. We then assessed the invasive capacity by a Matrigel in vitro invasion assay. The invasiveness of the IGFBP2-overexpressing cells was 1.8- to 2.9-fold greater than that of the vector-alone-transfected cells (*p *< 0.05; Figure 2C). Our tissue microarray findings were consistent with this finding. This suggests that acquisition of IGFBP2 is a very important step in the penetration of the extracellular matrix by ovarian cancer cells, which they may need to do before they can move to adjacent tissue and the lymphovascular space. This provides a site for occult progression or the formation of recurrent ovarian cancers. Indeed, ovarian cancer frequently spreads through the lymphatic system [27]. In fact, in one study, 20% of patients with early-stage invasive ovarian carcinoma whose tumors appeared to be confined to the ovary (stage I disease) were found to have lymph node metastasis [28]. The mortality rate in such patients is higher than that in patients without lymph node metastasis [29]. Our study therefore also provides strong evidence that IGFBP2 could be a factor indicating a poor prognosis.

### Disruption of IGFBP2 inhibits ovarian cancer cell invasion

**Figure 3:**
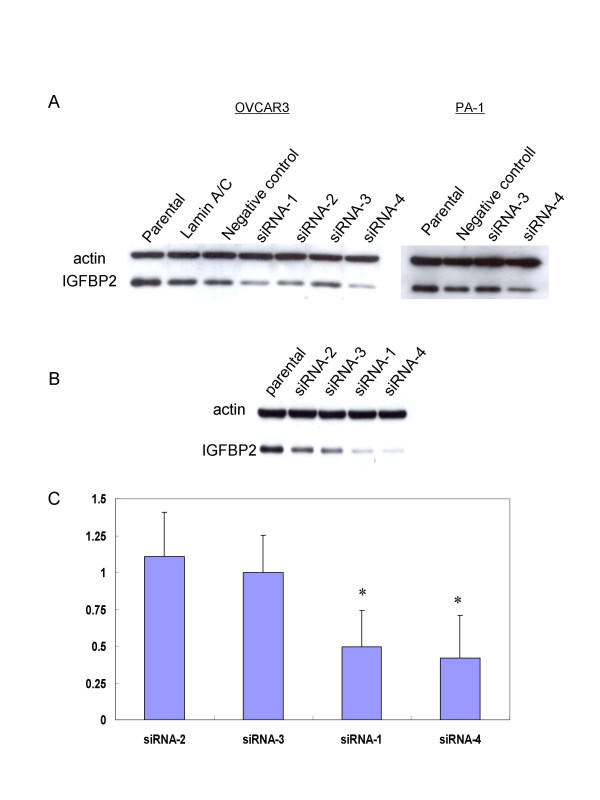
Attenuation of IGFBP2 inhibits ovarian cancer cell invasion. (A) Western blotting analysis of IGFBP2 after transfection of siRNA in OVCAR3 and PA-1. 4 siRNAs inhibit the IGFBP2 with various levels. IGFBP2 levels of siRNA-3 transfected cells have similar to those of Lamin A/C and negative control transfected cells. (B) Four different siRNAs were transfected to PA-1 ovarian cancer cells which has high endogenous IGFBP2. Different inhibiting levels of IGFBP2 were determined by western blotting analysis. siRNA-1 and -4 were working better than siRNA-2 and -3. (C) The invasion activity after 72 hours of siRNA transfection was significantly decreased in siRNA-1 and -4 treated cells comparing with siRNA-2 and -3 treated cells (*p < 0.05).

The observation that IGFBP2 increased the invasive capacity of ovarian cancer cells and those of previous studies prompted us to determine whether IGFBP2 could serve as a target of therapy for ovarian cancer. We used PA-1 ovarian cancer cells for this experiment because they express a high level of endogenous IGFBP2 and show a relatively high invasive capacity compared with other ovarian cancer cell lines. We designed siRNAs for four different target sites of IGFBP2 mRNA because not all siRNAs were expected to effectively attenuate IGFBP2 expression. Western blotting analysis showed that the IGFBP2 level after siRNA-3 transfection was comparable to the levels after the transfection of Lamin A/C and negative control siRNA in OVCAR3 and PA-1 ovarian cancer cell lines, suggesting that the siRNA-3 was not effective (Figure 3A). Considering that not all siRNA molecules work, this was not surprising. Therefore, we used siRNA-3-transfected cells as a negative control. siRNA-1 and siRNA-4 attenuated IGFBP2 expression in the PA-1 cells more than did either siRNA-2 or siRNA-3 (Figure 3B). Likewise, in the invasion assay, the invasive capacity of the cells transfected with siRNA-1 or siRNA-4 was significantly decreased compared with that of the cells transfected with siRNA-2 or siRNA-3 (*p *< 0.05; Figure 3C). These findings therefore support to the notion that IGFBP2 is a viable target of therapy for ovarian cancer.

## Conclusions

In this study, we showed that IGFBP2 was significantly overexpressed in invasive ovarian carcinomas compared with borderline ovarian tumors as well as normal ovarian tissues and that IGFBP2 increases invasion capability of ovarian cancer cells. These results provide evidence that IGFBP2 could promote ovarian cancer progression by augmenting the invasion potential. Although further investigations of molecular mechanisms are required, our findings using siRNA study support IGFBP2 as a novel target for the treatment of ovarian cancer.

## Methods

### Ovarian tissues and cell lines

Ovarian cancer and normal control tissues were obtained from The University of Texas M. D. Anderson Cancer Center tumor tissue bank with the approval of the Institutional Review Board. The ovarian cancer cell lines NIH:OVCAR3, SKOV3, OV-90, TOV-112D, TOV-21G, and PA-1 were purchased from the American Type Culture Collection (Manassas, VA). NIH:OVCAR3 cells were maintained in RPMI 1640 medium supplemented with 20% fetal bovine serum (FBS); SKOV3 and PA-1 cell were maintained in McCoy's 5a medium and Dulbecco's modified Eagle medium (DMEM)/F12, respectively, supplemented with 10% FBS; OV-90, TOV-112D, and TOV-21G cells were maintained in a 1:1 mixture of MCDB 105 medium and medium 199, supplemented with 15% FBS. All cells were kept at 37°C in a humidified atmosphere with 5% CO_2_. Media were routinely changed every 3 days.

### Construction of progression tissue microarray for ovarian serous tumors

Formalin-fixed, paraffin-embedded archival tissue blocks from ovarian cancer patients who had undergone surgery at The University of Texas M. D. Anderson Cancer Center between 1990 and 2001 were used to construct progression tissue microarrays according to previously described methods [30]. The progression tissue microarray consisted of normal ovarian surface epithelium from 6 individuals, serous borderline tumors from 17 patients, and invasive serous carcinomas from 40 patients. Tissue cores with a diameter of 1.0 mm were obtained from each sample and assembled into two separate tissue array blocks.

### Immunohistochemistry studies

Polyclonal antibody against IGFBP2 (c-18; Santa Cruz Biotechnology, Inc., Santa Cruz, CA) was used in the immunohistochemistry studies. This antibody is specific and does not cross-react with other isoforms of IGFBP. A standard indirect immunoperoxidase procedure (ABC-Elite; Vector Laboratories, Burlingame, CA) was used for all stains. In brief, antigen retrieval was performed by first placing unstained slides in a steamer for 25 min. The antibody against IGFBP2 (in a 1:1000 dilution) was overlaid on the tissue sections of tissue arrays, and incubation was performed at 4°C overnight. Secondary antibody incubation was performed at room temperature for 60 min. Mayer's hematoxylin nuclear stain was used as a counterstain. Staining intensity was graded on a 0–3 scale, where 0 = no staining as assessed by staining with anti-goat secondary antibody alone, 1 = weak (<10%), 2 = moderate (10–50%), and 3 = strong (50–100%). The results of the immunohistochemistry studies were statistically analyzed using a Mann-Whitney nonparametric U test.

### Stable clone establishment

To establish stable cell lines that overexpressed IGFBP2, we transfected SKOV3 ovarian cancer cell lines with a pcDNA3.1 expression vector encoding IGFBP2 cDNA using FuGENE6 reagent (Roche Diagnostics Corporation, Indianapolis, IN). Transfected cells were subsequently selected in the presence of G418 (300 μg/ml) for 5 weeks. The expression of IGFBP2 clones was determined from western blots of cell extracts with anti-IGFBP2 antibody (C-18). Two vector-transfected cell lines and three IGFBP2 stable cell lines were used in this study. Established stable cells were maintained without antibiotics.

### Western blot analysis

Equal amounts of proteins from the total cell lysates was separated by 10% SDS-PAGE and transferred electrophoretically to a Hybond ECL nitrocellulose membrane (Amersham Pharmacia Biotech, Chicago, IL). The membrane was blocked in 5% skim milk in 1× PBS and probed with a 1:1000 dilution of a goat polyclonal anti human IGFBP2 (C-18) overnight at 4°C. An enhanced chemiluminescence kit (ECL; Amersham Pharmacia Biotech, Piscataway, NJ) was used to visualize the proteins.

### In vitro chemoinvasion assay

We used 24-well BioCoat Matrigel invasion chambers (Becton Dickinson Labware, Bedford, MA) with an 8-μm pore polycarbonate filter coated with Matrigel to measure chemoinvasion. The lower compartment contained 0.75 ml of medium with 0.5% FBS as a chemoattractant. In the upper compartment, 5 × 10^4 ^to 2 × 10^5 ^cells/well were placed in triplicate wells and incubated for 22 h at 37°C in a humidified incubator with 5% CO_2_. After incubation, the cells that had passed through the filter into the lower wells were stained with Giemsa (Fisher Scientific, Orangeburg, NY) and counted by analyzing images under a microscope using software program as described previously [31]. All assays were repeated at least three times. Student's t-test was used to analyze the differences in the invasion rates between control cell lines and stable cell lines. A *p *value of <0.05 was considered statistically significant.

### siRNA transfection

Four different siRNA molecules designed for IGFBP2 mRNA and siRNA molecules for Lamin A/C and negative control were synthesized and purified (Qiagen, Valencia, CA). siRNA that targets Lamin A/C and siRNA that bears no homology with relevant human genes was used as a negative control.

AATGGCGATGACCACTCAGAA was the target sequence for siRNA1; AAGGGTGGCAAGCATCACCTT was the target sequence for siRNA2; AAGCGCCGGGACGCCGAGTAT was the target sequence for siRNA3; AACCTCAAACAGTGCAAGATG was the target sequence for siRNA4; AACTGGACTTCCAGAAGAACA was the target sequence for Lamin A/C; and AATTCTCCGAACGTGTCACGT was the target sequence for the negative control. siRNAs were dissolved in siRNA suspension buffer to a final concentration of 20 μM, and the mixture was heated to 90°C for 1 min and incubated at 37°C for 60 min. PA-1 ovarian cancer cells (2 × 10^5^) were plated to a 6-well plate and allowed to adhere for 24 h; the confluency of the cell monolayer at the time of transfection was 40–60%. 5 μg of siRNA and 15 μl of RNAiFect Transfection Reagent (Qiagen, Valencia, CA) was used. The cells were incubated under normal cell culture conditions. All assays were performed 72 h after treatment.

## Authors' contributions

EL carried out Western blotting analysis, establishing stable cells, invasion assay and siRNA transfection, participated in the analysis of all data and drafted the manuscript. CM and IS contributed to the statistical analysis. HW and JL provided frozen tissues and carried out tissue microarray and its analysis. AN performed the analysis of invasive assay. JK participated in its design of the study. JL and WZ participated in its design and coordination and helped draft the manuscript. All authors read and approved of the final manuscript.
